# Supplementary material for: XGBoost, A Novel Explainable AI Technique, in the Prediction of Myocardial Infarction: A UK Biobank Cohort Study
Source: Clin Med Insights Cardiol. 2022 Nov 8;16:11795468221133611. doi: 10.1177/11795468221133611 (PMC9647306; doi:10.1177/11795468221133611)
Supplement: sj-docx-1-cic-10.1177_11795468221133611 – Supplemental material for XGBoost, A Novel Explainable AI Technique, in the Prediction of Myocardial Infarction: A UK Biobank Cohort Study [file sj-docx-1-cic-10.1177_11795468221133611.docx]

**Supplementary Materials:

Logistic Regression:**

The goal of supervised regression models is to predict a target variable from a D-dimensional input vector. Linear regression models (such as the model implemented in this paper) will only use linear combinations of the input variables. In the case of two-class classification the target variable is written as a logistic sigmoid acting on a linear function of the input vector, maximum likelihood estimation is then used to determine the parameters of the logistic regression model^23^.

This paper used Scikit-learn’s implementation of a logistic regression model, with L2 regularization and ‘lbfgs’ solvers.
